# M2 macrophage microvesicle-inspired nanovehicles improve accessibility to cancer cells and cancer stem cells in tumors

**DOI:** 10.1186/s12951-021-01143-5

**Published:** 2021-11-27

**Authors:** Yuqi Wang, Xiang Gong, Jie Li, Hong Wang, Xiaoxuan Xu, Yao Wu, Jiaoying Wang, Siling Wang, Yaping Li, Zhiwen Zhang

**Affiliations:** 1 School of Pharmacy, Shenyang Pharmaceutical University, Shenyang, 110016 Liaoning China; 2 State Key Laboratory of Drug Research & Center of Pharmaceutics, Shanghai Institute of Materia Medica, Chinese Academy of Sciences, Shanghai, 201203 China; 3 Yantai Key Laboratory of Nanomedicine & Advanced Preparations, Yantai Institute of Materia Medica, Shandong, 264000 China

**Keywords:** Cancer stem cells, Macrophage, Microvesicle, Nanoparticles, Cabazitaxel

## Abstract

Cancer cells and cancer stem cells (CSCs) are the major players of cancer malignancy and metastasis, but they are extremely difficult to access. Inspired by the vital role of macrophages and microvesicle-mediated cell–cell communication in tumors, we herein designed M2 macrophage microvesicle-inspired nanovehicle of cabazitaxel (M-CFN) to promote accessibility to cancer cells and CSCs in tumors. In the 4T1 tumor model, M-CFN flexibly permeated the tumor mass, accessed cancer cells and CD90-positive cells, and significantly promoted their entry into CSC fractions in tumors. Moreover, M-CFN treatment profoundly eliminated aldehyde dehydrogenase (ALDH)-expressing CSCs in 4T1 and MCF-7 tumors, produced notable depression of tumor growth and caused 93.86% suppression of lung metastasis in 4T1 models. Therefore, the M2 macrophage microvesicle-inspired nanovehicle provides an encouraging strategy to penetrate the tumor tissues and access these insult cells in tumors for effective cancer therapy.

**Supplementary Information:**

The online version contains supplementary material available at 10.1186/s12951-021-01143-5.

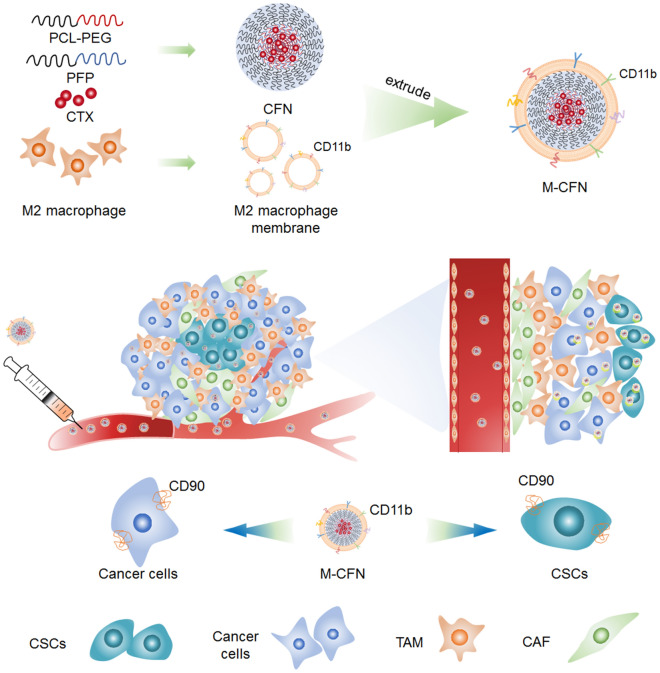

## Introduction

Cancer cells and cancer stem cells (CSCs) are the major players that orchestrate cancer malignancy and metastasis in many types of solid tumors [1, 2]. Cancer cells are a subgroup of cells that are heterogeneously distributed in tumor tissues, and CSCs are a small subpopulation of cancer cells with principal features of self-renewal, proliferation and differentiation, which is only 0.05–3% of total cancer cells in tumors [3–5]. Cancer cells and CSCs usually undergo a dynamic reciprocal transformation process [6, 7]. CSCs can transform into rapidly dividing cancer cells to foster tumor progression, and cancer cells can evolve into CSCs to protect themselves from therapeutic intervention. Some commonly used anticancer drugs, such as paclitaxel, cisplatin, and sunitinib, efficiently damage rapidly dividing cancer cells [1, 8, 9], but they are ineffective in eliminating CSCs; worse still, they may unexpectedly enrich the ratio of CSCs in tumors [9–11]. To date, only a few therapeutic agents have been used alone to directly eliminate CSCs or in combination with modulatory agents to sensitize them to standard therapies [1, 12–19]. However, cancer cells and CSCs are frequently embedded in the dense tumor stroma comprising versatile stromal cells and extracellular matrix networks, making them extremely difficult access [5, 20, 21]. Rationally, therapeutic agents should be delivered across the tumor stroma barriers and access cancer cells and CSCs to exert anticancer effects [22, 23]. Although nanovehicles hold great potential for accumulating at tumor sites, they are often trapped around the tumor vasculature and ineffective in penetrating the tumor tissues, drastically restricting their access to cancer cells and CSCs in tumors [24]. Therefore, it is impressively desired to develop novel delivery strategies to improve their accessibility to these insult cells for cancer therapy.

Cancer cells are usually embedded in the tumor stroma and surrounded by a variety of accessory cells, such as cancer-associated fibroblasts (CAFs), tumor-associated macrophages (TAMs) and endothelial cells etc. [25–27]. CSCs are believed to reside in CSC niches comprised of versatile accessory cells, extracellular matrix (ECM) components, and networks of cytokines and growth factors [6, 28, 29]. Of note, TAMs, usually M2-phenotype macrophages in tumors, are the major supportive cells in tumor microenvironments and CSC niches, that play a vital role in promoting cancer cell proliferation, metastasis and maintaining the CSC state [2, 30–32]. Compelling evidence has revealed that macrophages can interact with breast cancer CSCs via a variety of mechanisms to support CSC niches, such as the binding of CD11b to CD90 markers [33–35]. Interestingly, TAMs can communicate with cancer cells or CSCs in tumors via extracellular vesicles such as microvesicles or exosomes [29, 36–41], which provides a substantial opportunity to diffuse across the tumor stroma and access cancer cells and CSCs in tumors. Moreover, due to the possible immunogenicity of the macrophages from a different donor, autologous macrophages have been used in clinical trials to treat a variety of patients with tumors or other diseases, and have been validated to be safe in Phase I clinical trials for liver cirrhosis [42–45]. To date, few if any reports of M2-macrophage microvesicles have been explored as delivery vehicles to improve drug accessibility to these insult cells in tumors.

**Scheme 1:**
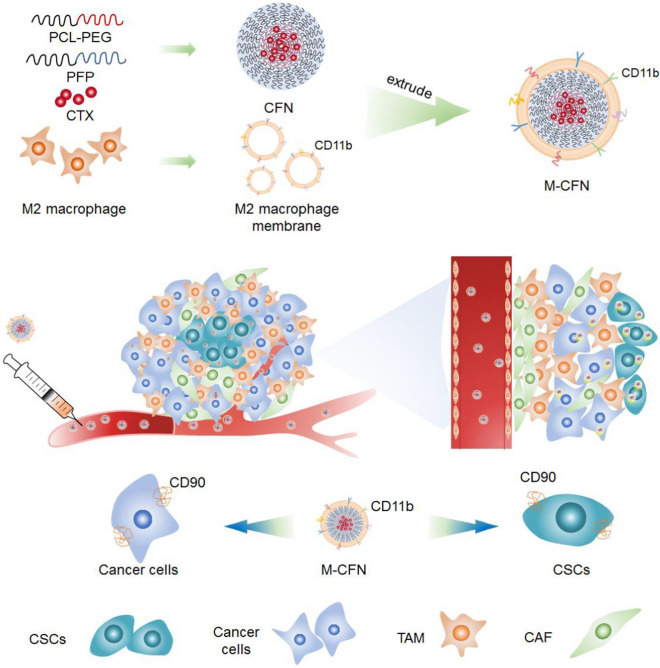
Schematic illustration of M2-macrophage microvesicle-inspired nanovehicles (M-CFN) penetrating tumor tissues and preferentially accessing cancer cell and CSC fractions for effective cancer therapy

Based on this rationale, we herein designed a M2-macrophage microvesicle-inspired nanovehicles of cabazitaxel (CTX) (M-CFN) to promote their accessibility to cancer cells and CSCs in tumors to promote effective cancer therapy (Scheme 1). CTX, a chemotherapeutic drug with poor affinity to the efflux transporter P-glycoprotein, was selected as the therapeutic agent to kill cancer cells and CSCs [46, 47]. M-CFN was developed by camouflaging cabazitaxel-loaded polyfluorocarbon nanoparticles (CFN) with M2-macrophage membranes to endow them with macrophage microvesicle-mimic features, thereby promoting their intratumoral permeation and improving accessibility to the cancer cell and CSC fractions in tumors. We investigated the capacity of M-CFN to promote tumor penetration and access cancer cells and CSCs in 4T1 tumors. Moreover, we measured the therapeutic benefits of M-CFN and its effects on eradicating CSCs in 4T1- and MCF-7- tumor models.

## Materials and methods

### Materials

Poly(maleic anhydride-alt-1-octadecene) (30,000–50,000 Da) (PMO) was provided by Sigma-Aldrich (St. Louis, MO, USA) and 1H,1H-perfluorooctylamine was obtained from Tokyo Chemical Industry Co., Ltd (Tokyo, Japan). Methoxypolyethylene glycol 5000 amine (mPEG-NH_2_) (5000 Da) was supplied by Seebio Biotech (Shanghai) Co., Ltd. (Shanghai, China). Amphiphilic polyfluorocarbon polymeric materials (PFP) were synthesized by grafting mPEG-NH_2_ and perfluorooctylamine to PMO and were further characterized using ^1^H-NMR and fluorine spectra (Additional file 1: Figs. S1, S2). Poly(ε-caprolactone)-block-poly(ethylene glycol) (PCL-PEG) was purchased from Shanghai Leon Chemical Ltd. (Shanghai China). CTX was provided by Dalian Meilun Biotechnology Co. Ltd. (Dalian, China).

Murine 4T1 breast cancer cells, RAW 264.7 cells and human MCF-7 breast cancer cells were provided by the Cell Bank of Shanghai, Chinese Academy of Sciences (CAS, Shanghai, China). 4T1 cancer cells with stable expression of green fluorescence proteins (4T1-GFP) were supplied by Sciencelight Biology Science and Technology Co., Ltd (Shanghai, China). 4T1 and 4T1-GFP cells were incubated with RPMI-1640 media containing 10% FBS (Gibco) at 37 °C and 5% CO_2_ in a humidified incubator, while RAW 264.7 and MCF-7 cells were cultured using Dulbecco's modified Eagle medium (DMEM) with 10% FBS (Gibco) under the same conditions.

Female BALB/c mice (18–22 g) and BALB/C-nu nude mice (18–22 g) were obtained from the Shanghai Experimental Animal Center, CAS (Shanghai, China) to develop tumor models for further measurements. To develop the 4T1- or 4T1-GFP- induced tumor model, approximately 1,000,000 cells were injected into the second mammary pad of BALB/C mice. To induce the MCF-7 breast cancer model, approximately 1 × 10^7^ cells were subcutaneously injected into BALB/C-nu mice. When the tumor size reached approximately 200 mm^3^, the tumor mass was collected, cut into small pieces and implanted into the second mammary pads of BALB/C-nu mice for further measurements. These animal experiments were performed according to protocols approved by the Institutional Animal Care and Use Committee of Shanghai Institute of Materia Medica (SIMM), CAS.

### Preparation of M-CFN

M-CFN was prepared in two steps: (i) preparation of CFN, and (ii) isolation of M2 macrophage membranes and camouflaging them onto CFN to form M-CFN.

CFN was prepared with PFP, PCL-PEG and CTX (10:10:1, w/w) using an emulsion-evaporation technique. In brief, these ingredients were dissolved in trichloromethane, dropped into double-distilled water at a 1:5 volume ratio and then emulsified using a sonication probe (JYD-650L, Shanghai, China). Then, the emulsion was evaporated under reduced pressure (B-480, Buchi, Switzerland) to remove trichloromethane to obtain CFN. The CTX concentration in CFN solution was approximately 1.0 mg/mL.

M2 macrophages were induced from RAW 264.7 cells by incubation with 40 ng/mL interleukin 4 (IL-4) (PeproTech, USA) in DMEM for 48 h. Then, the phenotype of the induced macrophages was examined by flow cytometry analysis (FACSCalibur, BD Biosciences) after incubation with anti-CD86-APC and anti-CD206-PE antibodies according to the manufacturer’s protocols. M2 macrophages were denoted as CD86^−^CD206^+^ cells. Thereafter, the induced M2 macrophages were collected to isolate the macrophage membranes according to our previously described method [48].

M-CFN was prepared by camouflaging CFN with a macrophage membrane using an extrusion method. In brief, 1 mL of CFN (CTX, 1.0 mg/mL) was mixed with macrophage membranes from approximately 4 × 10^7^ cells and extruded through a series of carbonate films with pore sizes of 800 nm, 400 nm and 200 nm 20 times to obtain M-CFN.

### Characterization of CFN and M-CFN

The morphologies of CFN and M-CFN were determined using transmission electron microscopy (TEM, 120 kV, Talos, FEI, USA) after negative staining with saturated uranyl acetate solution. The size distribution and zeta potential values were measured using dynamic light scattering (DLS) analysis on a Malvern Nano ZS90 analyzer (Malvern, UK).

The encapsulation efficiency (EE) values of CTX in CFN and M-CFM were determined using high performance liquid chromatography (HPLC) analysis. The unentrapped CTX was separated from the CFN or M-CFN by centrifugation using an ultrafiltration tube (10 KDa) at 8000*g* for 20 min. The drug levels in the total solution and filtration were determined by HPLC analysis (Waters, USA) under the following conditions: column, XBridge C18 Column (250 mm × 4.6 mm, 5 μm i.d., Waters); mobile phase, acetonitrile–water (70:30, v/v); flow rate, 1.0 mL/min; detection wavelength, 254 nm. The EE values (%) were defined as the ratio of the amount of entrapped drug compared to the total drug amount in the nanovehicles.

To evaluate their stability in the mimicked physiological fluids, CFN and M-CFN were incubated with phosphate buffered saline (PBS, pH 7.4) at room temperature for 24 h and the particle size was determined using the Nano ZS90 analyzer at predetermined time points. Moreover, CFN and M-CFN were incubated in PBS (pH 7.4) and complete fetal bovine serum (FBS) at 37 °C for 30 h. At certain time intervals, samples were collected, and EE values were monitored by HPLC analysis as described above to characterize the percentage of drug remaining in the nanovehicles.

To confirm whether the macrophage membrane (MM) preserves the microvesicle features of macrophages, microvesicles were isolated from the serum-free culture media of M2 macrophages as previously described [49]. Meanwhile, to confirm the camouflaging of MM onto CFN in M-CFN, the proteins from M2 macrophages, MM, macrophage microvesicles, M-CFN and CFN macrophage membranes and M-CFN were extracted using RIPA lysis buffer (moderate, Beyotime, China) for western blotting assays. The typical protein markers CD11b, CSF1R, α4, β1 and CCR2 in these samples were measured using primary antibodies against CD11b (Abcam, ab184308, 1:1000), CSF1R (Affinity, AF0080, 1:500), α4 (Affinity, DF6135, 1:500), β1 (Affinity, AF5379, 1:500) and CCR2 (Affinity, DF2711, 1:1000) followed by horseradish peroxidase (HRP)-labeled secondary antibodies (Simuwu, SD0039, 1:40000). In contrast, the counterpart CFN without membrane decoration was used as a negative control. In addition, the expression of CD11b in M2 macrophages was confirmed by flow cytometry analysis.

### In vitro cellular uptake in parent 4T1 cancer cells and 3D tumorspheres

The cellular uptake of CFN and M-CFN was measured in parental 4T1 cells and 4T1-induced 3D tumorspheres using confocal laser scanning microscopy (CLSM) (TCS-SP8 STED, Leica, Germany) and fluorescence spectrum analysis (EnSpire, PerkinElmer, Singapore). CFN and M-CFN were labeled with a hydrophobic probe of DiIC18(3) (DiI) by physical encapsulation for the measurements.

The parent 4T1 cancer cells were cultured in RPMI 1640 media as described above. To develop CSC-enriched 3D tumorspheres, 4T1 cells were cultured in ultralow attachment plates with serum-free DMEM/F12 media containing 5 mg/L insulin, 1 × B27, 0.4% (w/v) bovine albumin, 20 ng/mL epidermal growth factor (EGF), 20 ng/mL basic fibroblast growth factor (bFGF), 100 U/mL penicillin, and 100 µg/mL streptomycin. After 7 days of culture, tumorspheres were formed and used for further measurements. Expression of CD90 markers in CSC-enriched 3D tumorspheres and parent 4T1 cancer cells was investigated using immunofluorescence assays with anti-CD90 primary antibody (Affinity, DF6849, 1:200) followed by Alexa Fluor 488-labeled goat-anti-rabbit IgG (H + L) (Yeasen, 33106ES60, 1:200).

To visualize their uptake into parental 4T1 cells under CSLM, cells were seeded into a 24-well plate using a round coverslip at 50, 000 cells/well and cultured overnight. DiI-labeled CFN and M-CFN were added to each well at 1 µg/mL DiI, and incubated for 4 h. Then, the cells were stained with Hoechst 33342 for visualization under CSLM. To determine their uptake in CSC-enriched 3D tumorspheres, the tumorspheres were incubated with DiI-labeled CFN and M-CFN at 1 µg/mL DiI for 4 h, and counterstained with Hoechst 33342 for CLSM detection. For quantification, the parent 4T1 cells and tumorspheres were incubated with DiI-labeled CFN and M-CFN at 1 µg/mL DiI for 4 h and then harvested for quantification by fluorescence spectrum analysis (EnSpire, PerkinElmer, Singapore).

To determine the possible mechanism of M-CFN-mediated preferential uptake in CSC-enriched 3D tumorspheres, DiI-labeled M-CFN was pretreated with anti-CD11b (eBioscience, 14-0112-82, 1:50) at 4 °C overnight. Then, M-CFN and anti-CD11b-pretreated M-CFN were incubated with CSC-enriched 3D tumorspheres for 4 h, and quantified by flow cytometry analysis.

### Cytotoxicity in parent 4T1 cancer cells and CSC-enriched 3D tumorsphere cells

The cytotoxicity of CFN and M-CFN was measured in the CSC-enriched 4T1 tumorspheres and parental 4T1 cancer cells. To evaluate cytotoxicity in CSC-enriched tumorsphere cells, tumorspheres that were developed from 5000 cells in 24-well ultralow attachment plates were treated with free CTX, CFN and M-CFN at CTX concentrations ranging from 5 ng/mL to 50 μg/mL or comparable doses. After 5 days of incubation, the cell viability from each group was measured using a cell counting kit-8 (CCK8). In contrast, to evaluate the cytotoxicity in parental 4T1 cells, cells were seeded into 96-well plates at 3,000 cells/well and incubated overnight. Then, free CTX, CFN and M-CFN were added to each well with CTX ranging from 5 ng/mL to 50 μg/mL or comparable doses and incubated for an additional 48 h. Cells without any treatment were used as a negative control. The cell viability from each treatment was measured using the CCK8 assay kit on a microreader analyzer (EnSpire, PerkinElmer, Singapore).

### Effects on inhibiting tumorsphere formation and eliminating ALDH-positive CSCs

The effects of M-CFN on inhibiting tumorsphere formation and eliminating ALDH-positive CSCs were measured in 4T1 and MCF-7 cancer cell models. In brief, 4T1 cells or MCF-7 cells were pretreated with free CTX, CFN or M-CFN at 0.25 μg/mL CTX for 12 h. Cells without any treatment were used as controls. Then, these cells were collected and seeded into 6-well ultralow attached plates at 40,000 cells/well, and incubated with the aforementioned serum free DMEM/F12 culture media for 7 days to form the tumorspheres. The tumorspheres from each treatment were imaged under an inverted microscope (IX81, Olympus, Japan). Thereafter, the tumorspheres from each group were collected, dissociated into single cells and stained using an Aldefluor™ kit (#01700, STEMCELL Technologies). The proportion of ALDH-positive CSC fractions in tumorspheres was monitored using flow cytometry analysis (FACSCalibur, BD, USA).

### In vivo tumor accumulation, permeation and cancer cell access

The in vivo tumor accumulation of M-CFN and CFN was measured in 4T1-induced tumor models. For in vivo imaging, CFN and M-CFN were labeled with a hydrophobic dye of DiIC18(7) (DiR) by physical encapsulation. When the tumor size reached approximately 100 mm^3^, DiR-labeled CFN and M-CFN were injected into tumor-bearing mice via the tail vein at 1.0 mg/kg DiR. At predetermined time points, mice were anesthetized and the fluorescence signals of DiR from these two groups were monitored using an in vivo imaging system (IVIS Spectrum, PerkinElmer, USA). At 8.0 h postinjection, mice were necropsied, and the major organs including the heart, liver, spleen, lung, kidney and tumor from each group were collected and imaged using the in vivo imaging system.

To quantify the drug distribution in the major organs, tumor-bearing mice were intravenously administered free CTX, CFN and M-CFN at 10 mg/kg CTX (n = 3). Eight hours after injection, mice were necropsied, and the major organs from each treatment were collected, homogenized with 0.5 mL of dimethyl sulfoxide, and centrifuged at 10,000*g* for 10 min. The drug concentration in the supernatant was determined using HPLC analysis as described above.

To investigate the intratumoral permeation behaviors, DiI-labeled CFN and M-CFN were intravenously administered to tumor-bearing mice at 1.0 mg/kg DiI. Eight hours after injection, mice were necropsied and the tumor tissues were collected for cryostat sectioning at 10 µm (CM1950, Leica). Afterward, the sections were counterstained with DAPI for visualization under CLSM (TCS-SP8 STED, Leica, Germany). The fluorescence signals of DiI in the entire tumor sections were documented to outline their intratumoral distribution. The captured images were analyzed using ImageJ software (National Institutes of Health, Bethesda, MD, USA) for better clarification.

To evaluate their accessibility to the cancer cell fraction in tumors, the DiI-loaded CFN and M-CFN were injected into 4T1-GFP-induced tumor models via the tail vein at 1.0 mg/kg DiI. At 8.0 h postinjection, the tumor tissues were removed, embedded in frozen media and sectioned at 10 µm on a cryotome (Leica 1950, Germany). The sections were stained with DAPI for measurement. The green signals of cancer cell clusters, red signals of these two nanovehicles and the blue signals of nuclei were recorded to evaluate their accessibility to the cancer cell regions in tumors. The images were further analyzed using the ImageJ software.

To measure their access to CD90-expressing cancer cells and CSCs in 4T1 tumors, the tumor sections were treated with a primary antibody against CD90 (Affinity, DF6849, 1:200) followed by Alexa Fluor 488-labeled goat anti-rabbit IgG (H + L) (Yeasen, 1:200) for CLSM detection. The access of nanovehicles to the CD90-positive regions in tumors was denoted as the colocalization of these two fluorescence signals, which was further analyzed using the ImageJ software. Moreover, the access of CFN and M-CFN to CD90-expressing cells in tumors was determined using flow cytometric analysis. In brief, tumor tissues from DiI-labeled CFN- and M-CFN-treated groups were collected, cut into small pieces and treated with digestion buffer containing 0.2 mg/mL collagenase type IV (Yeasen, 40510ES60), 0.2 mg/mL hyaluronidase (Yeasen, 20426ES60) and 0.1 mg/mL dexoyridonuclease I (Yeasen, 10608ES25) at 37 °C for 2 h. The cell suspensions were collected by centrifugation, and then incubated with PE-anti-CD90 (Biolegend, 202,523, 1:50) at 4 °C for 1 h. The uptake of CFN or M-CFN into CD90-expressing cells was determined using flow cytometry.

### In vivo access to CSC fractions in tumor

The in vivo access of CFN and M-CFN to the CSC fractions in tumors was measured in 4T1-induced tumor models. DiI-labeled CFN and M-CFN were intravenously administered to tumor-bearing mice at 1.0 mg/kg DiI. At 8.0 h postinjection, the tumor tissues were removed, frozen and sectioned at 10 µm (Leica 1950, Germany). The tumor sections were stained with primary antibodies against ALDH1A1 (Abcam, ab52492, 1:100), SOX2 (Abcam, ab97959, 1:1000) and Oct4 (Abcam, ab200834, 1:50), followed by Alexa Fluor 488-labeled IgG (H + L) secondary antibody (Beyotime, Jiangsu, China). In contrast, these sections were stained with DAPI for CLSM detection. The access of nanovehicles to these CSC fractions were denoted as the overlap of the red fluorescence signals of nanovehicles and the green signals of typical markers of CSCs in tumor sections. These images were analyzed using Image-Pro Analyzer 3D 7.0 (Media Cybernetics, MD, USA) to monitor the colocalization of nanovehicles with CSCs in tumors and were analyzed using ImageJ software to evaluate the access of nanovehicles to CSC fractions in tumors.

### In vivo therapeutic efficacy

The in vivo therapeutic efficacy of M-CFN was measured in a 4T1-induced metastatic breast cancer model, which was developed as described above. When the tumor volume reached approximately 100 mm^3^, the tumor-bearing mice were intravenously administered PBS control, MM, free CTX, CFN and M-CFN at 10 mg/kg CTX or comparable doses every three days for a total of six injections (n = 5). The tumor size and body weights from each group were monitored. After six cycles of injection, mice were necropsied and the tumor tissues from each treatment were collected, imaged and accurately weighed to calculate the tumor inhibition effects. To assess apoptosis in tumors, tumor tissues were subjected to TUNEL assays. Concurrently, the lungs from each treatment were collected and the visualized metastatic nodules in each lung were counted to calculate the inhibitory effects on lung metastasis. Furthermore, histological examinations of the lung tissues from each treatment were performed using hematoxylin and eosin (H&E) staining to detect metastatic foci in the lungs.

To confirm the therapeutic benefits of M-CFN, we evaluated antitumor efficacy in an MCF-7-induced tumor model. When the tumor size reached 100 mm^3^, tumor-bearing mice were injected with PBS control, free CTX, CFN or M-CFN at 5 mg/kg CTX on days 1 and 6 for a total of two injections. During the treatments, the tumor volume and body weight changes were recorded. When the tumor volume in the PBS group was approximately 1000 mm^3^, the experiments were terminated, and the tumor tissues from each treatment were collected, imaged and weighed to evaluate the inhibitory effects on tumor growth.

### In vivo effects on eradicating ALDH-expressing CSC fractions in tumors

To detect the CSC-eradicating ability of M-CFN, the tumor tissues from each group in 4T1- and MCF-7 tumor models were embedded in paraffin and sectioned at 5 µm. The sections were successively incubated with anti-ALDH1A1 (Abcam, ab52492, 1:100) and Alexa Fluor 488 labeled IgG (H + L) (Beyotime, Jiangsu, China). In contrast, the nuclei were stained with DAPI and monitored using a pannoramic scan (Pannoramic MIDI, 3D Histech, Hungary). The ALDH-positive (ALDH^high^) CSC fractions in tumors are denoted as green fluorescence signals. The captured images were analyzed using ImageJ software for quantification.

### Statistical analysis

Data are expressed as the mean standard deviation (SD) and were analyzed using Student’s *t*-test. Statistical differences were significant at *p < 0.05 and very significant at **p < 0.01.

## Results and discussion

### Preparation and characterizations of M-CFN

**Fig. 1:**
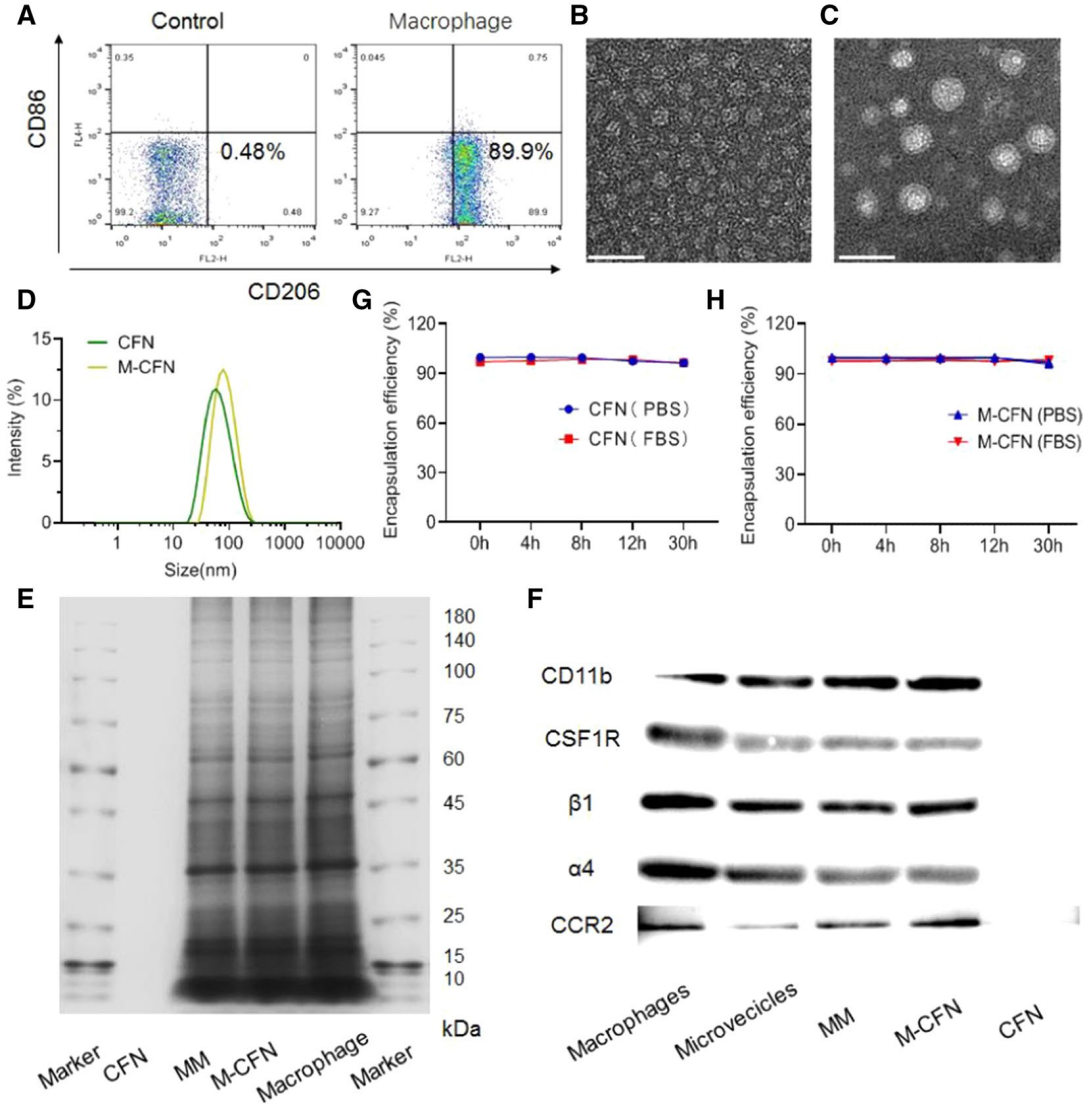
Characterizations of M-CFN. **A** Characterization of M2 macrophages by flow cytometry examinations. **B** TEM images of CFN, scale bar, 50 nm. **C** TEM images of M-CFN, scale bar, 50 nm. **D** Particle size distribution of CFN and M-CFN measured by DLS analysis. **E** Protein profiles in CFN, macrophage membrane (MM), M-CFN and macrophages by electrophoresis assays. **F** Typical protein markers expressed in macrophages, microvesicles, MM, M-CFN and CFN by western-blot analysis. **G** EE values of CTX in CFN upon incubation in PBS (pH 7.4) and FBS. **H** EE values of CTX in M-CFN upon incubation in PBS (pH 7.4) and FBS

Initially, M-CFN was fabricated in two steps: (1) preparing CFN with CTX and amphiphilic polyfluorocarbon polymeric materials (PFP) and poly(ε-caprolactone)-block-poly(ethylene glycol) (PCL-PEG); (2) inducing M2 macrophages from murine RAW264.7 cells and isolating M2 macrophage membranes to camouflage CFN via an extrusion method to form M-CFN. Details of the synthesis and characterization of PFP are provided in the Additional file 1: Figs. S1, S2. M2 macrophages were induced by incubating RAW264.7 cells with 40 ng/mL IL-4 for 48 h and characterized by flow cytometry analysis, which were denoted as CD86^−^CD206^+^ cells (Fig. 1A). The TEM images indicated that both CFN and M-CFN were nanosized spherical particles with a size less than 50 nm (Fig. 1B, C). In contrast to CFN, M-CFN displayed a typical core–shell nanostructure (Fig. 1C). When CFN was decorated with the isolated macrophage membrane, the average hydrodynamic diameter increased from 54.79 ± 1.50 nm for CFN to 73.59 ± 1.92 nm for M-CFN (Fig. 1D). Meanwhile, the zeta potential values were altered from 0.74 ± 0.25 mV for CFN to -9.24 ± 1.93 mV for M-CFN. Moreover, the protein profiles in macrophages, macrophage membranes (MM), CFN and M-CFN were determined by electrophoresis assays. As shown in Fig. 1E, no protein signals were detected in CFN, but the major protein compositions in macrophage membrane were largely retained in M-CFN. Meanwhile, western blotting analysis indicated that typical markers of macrophages, including CD11b, colony stimulating factor 1 receptor (CSF1R), β1-integrins, α4-integrins and C–C motif chemokine receptor 2 (CCR2), were readily detected in the macrophage derived microvesicles, MM and M-CFN, but were undetectable in their counterpart CFN (Fig. 1F). These data indicated that MM preserves the typical markers of macrophage microvesicles, and MM was effectively camouflaged onto CFN in the M-CFN system, which may endow them with macrophage microvesicle-mimicking features. In addition, the expression of CD11b, a surrogate marker of macrophages that recognizes CSCs in tumors, was also confirmed by flow cytometry analysis (Additional file 1: Fig. S3). Increasing data have shown that extracellular vesicles play a key role in tumor progression and could be used as effective delivery vehicles for targeted drug delivery [50, 51]. Concurrently, TAMs can effectively communicate with CSCs or cancer cells in tumors with extracellular vesicles such as microvesicles or exosomes [2, 6, 36]. Accordingly, the combination of nanosized vehicles and macrophage microvesicle-mimicking MM in the M-CFN system provides a substantial opportunity to improve accessibility to CSCs or cancer cells in tumors. Although the CD11b marker is constitutively expressed in both M1 and M2 macrophages, the highly expressed markers CSF1R, CCR2 and α4β1 integrins on the M2 macrophage surface facilitate their accumulation at tumor sites and promote their accessibility to cancer cells and CSCs in tumors [52].

Then, we investigated the EE of CTX in CFN and M-CFN using HPLC analysis. The measured results indicated that the EE value of CTX was 99.79 ± 0.01% for CFN and 99.64 ± 0.11% for M-CFN, indicating the high encapsulation of CTX in these two nanoparticles. Meanwhile, upon incubation in phosphate buffered saline (PBS, pH 7.4) or fetal bovine serum (FBS), the EE values of CTX in CFN and M-CFN barely changed with incubation time (Fig. 1E, F). After 30 h of incubation, over 95% of CTX remained in CFN and M-CFN. In addition, when they were incubated in PBS (pH 7.4) for 24 h, no changes in the mean diameter of CFN and M-CFN were detected (Additional file 1: Fig. S4). These data confirmed their good stability in mimicked physiological fluids, suggesting their feasibility for in vivo delivery.

### Cellular uptake and CSCs-eradicating efficacy

**Fig. 2:**
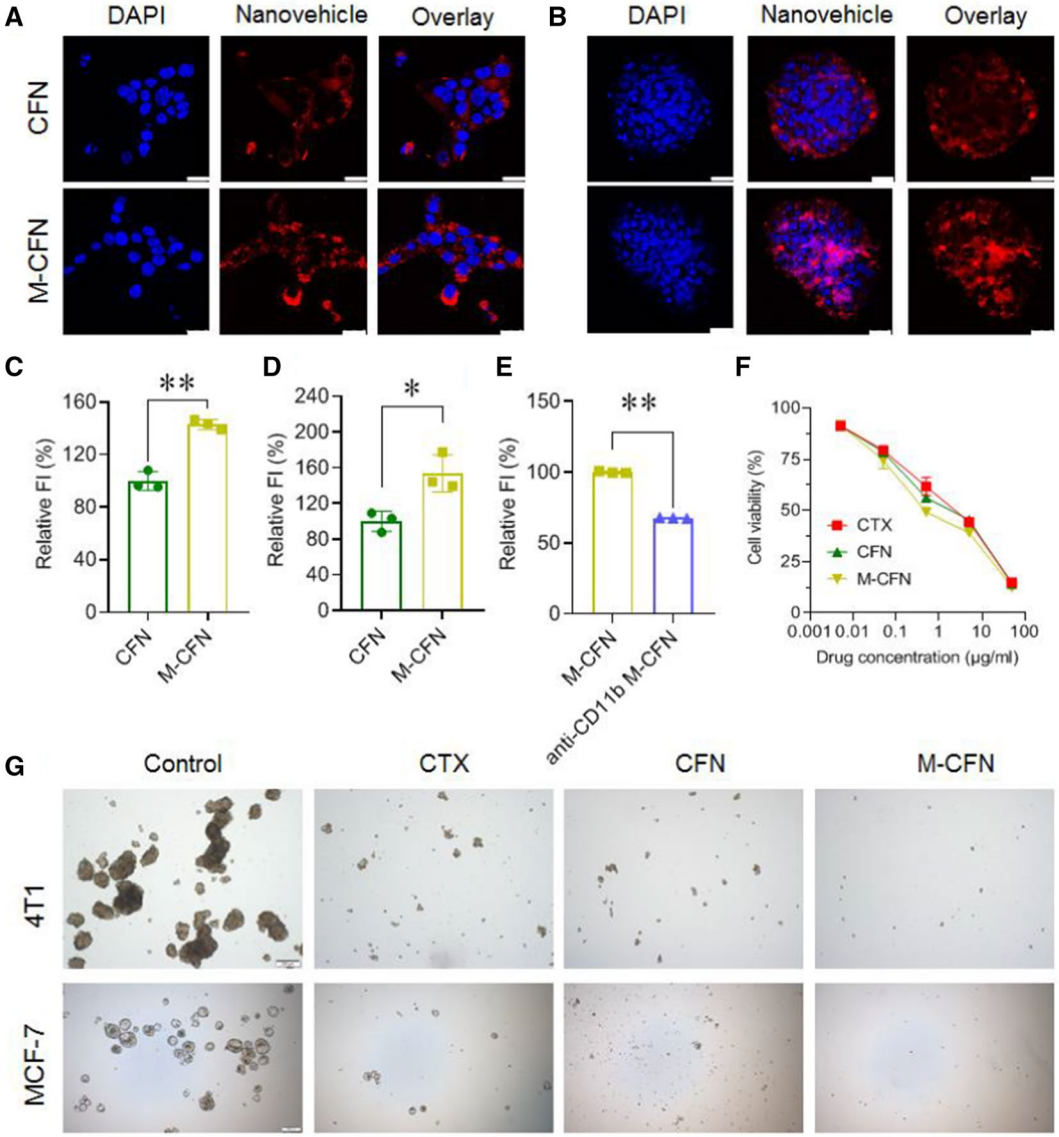
In vitro cellular uptake and inhibition of tumorsphere formation. **A** Cellular uptake of CFN and M-CFN in parental 4T1 cancer cells, scale bar, 25 μm. **B** Cellular uptake of CFN and M-CFN in 4T1-induced tumorspheres, scale bar, 25 μm. **C** Quantified cellular uptake in 4T1 cancer cells, **p < 0.01. **D** Quantified cellular uptake in CSC-enriched tumorsphere cells, *p < 0.05. **E** Uptake of M-CFN and M-CFN + antiCD11b in 4T1-induced tumorsphere cells. **F** Cytotoxicity in CSCs-enriched tumorsphere cells. **G** Effects on inhibiting tumorsphere formation of 4T1 and MCF-7 cancer cells, scale bar, 200 μm

The cellular uptake of M-CFN was determined in comparison to CFN in CSC-enriched 3D tumorspheres and parental 4T1 cancer cells using confocal laser scanning microscopy (CLSM) and fluorescence spectra analysis. The results indicated that M-CFN was effectively internalized into both parent 4T1 cells and CSC-enriched tumorspheres with a fluorescence intensity stronger than that of CFN (Fig. 2A, B). Compared with CFN, the cellular uptake of M-CFN increased 1.43-fold in parental 4T1 cancer cells and 1.54-fold in CSC-enriched tumorspheres (Fig. 2C, D). CD11b expressed on macrophages is crucial for their binding to the CD90 markers of breast cancer CSC [2]. Expression of CD90 in CSC-enriched 4T1 tumorsphere cells and parental 4T1 cancer cells was verified by immunofluorescence imaging, which could interact with CD11b in macrophages (Additional file 1: Fig. S5). To investigate the role of CD11b in M-CFN preferential uptake, we blocked CD11b on the M-CFN particles with anti-CD11b antibodies and measured their uptake in the CSC tumorsphere model. Compared to M-CFN, the uptake of anti-CD11b treated M-CFN was significantly reduced by 32.50% (Fig. 2E), indicating the important role of CD11b in the preferential cellular uptake of M-CFN in CSC-enriched tumorsphere cells.

**Fig. 3:**
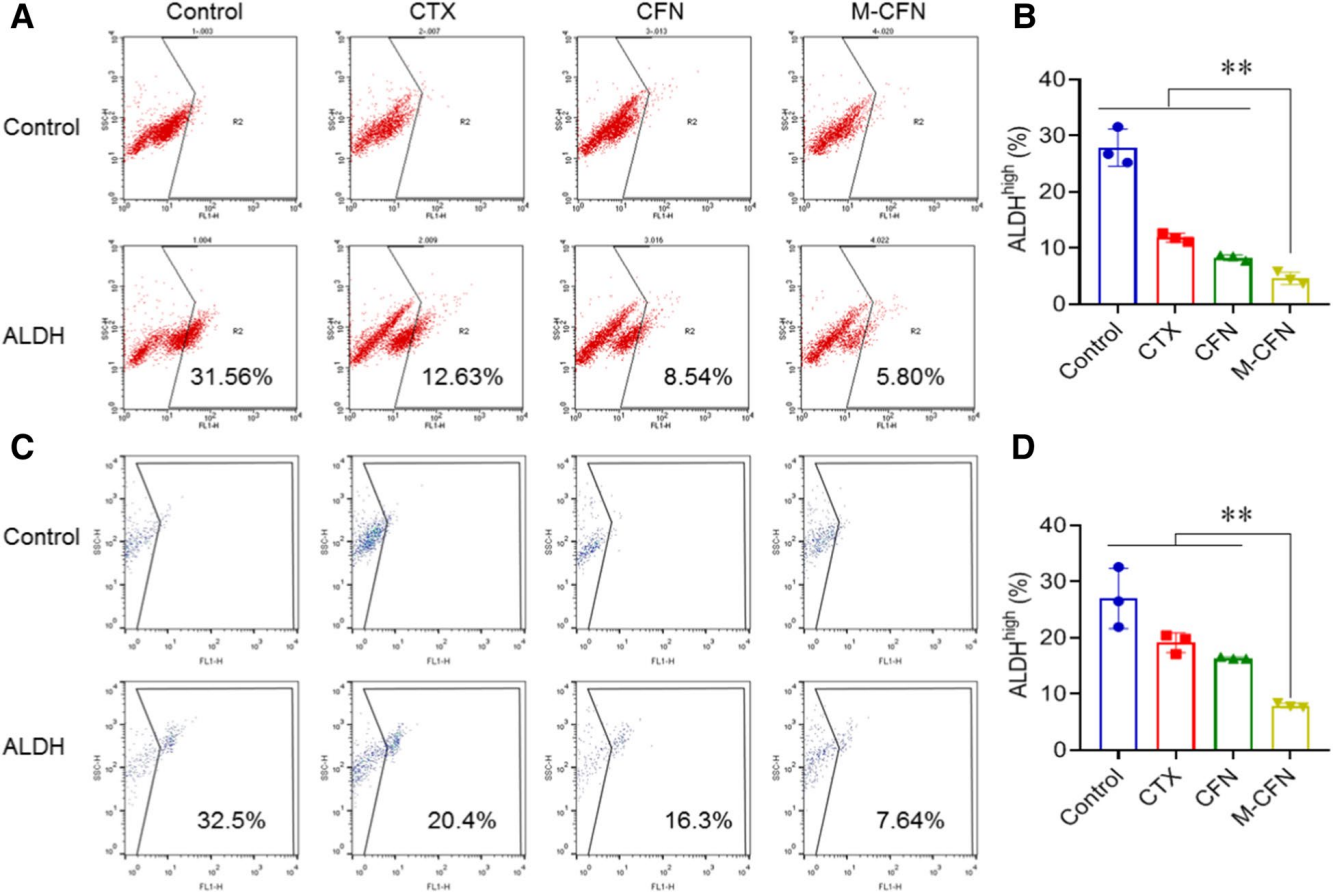
Effects of M-CFN on eradicating ALDH-positive CSC fractions in tumorspheres. **A** Typical flow cytometry profiles of ALDH-positive CSCs in 4T1-induced tumorspheres from each treatment. **B** Quantified results of M-CFN in eradicating ALDH-positive CSCs in 4T1-induced tumorspheres, **p < 0.01. **C** Typical flow cytometry profiles of ALDH-positive CSCs in MCF-7-induced tumorspheres. **D** Quantified results of M-CFN in eradicating ALDH-positive CSCs in MCF-7-induced tumorspheres, **p < 0.01

In the CSC-enriched 4T1 tumorsphere cells and parental 4T1 cancer cells, free CTX, CFN and M-CFN exhibited considerable cytotoxicity in a concentration-dependent manner (Fig. 2F, Additional file 1: Fig. S6). Then, we evaluated the effect of M-CFN on inhibiting the self-renewal ability of 4T1 and MCF-7 breast cancer cells using a tumorsphere-forming assay. The residual 4T1 or MCF-7 cancer cells from the CTX-, CFN- and M-CFN- (0.25 µg/mL CTX or comparable concentrations)-treated groups were cultured in ultralow attachment plates for tumorsphere-formation (Fig. 2G). After 7 days, typical large tumorspheres were clearly observed in the control group, but only a few small cell spheres were detected in the CTX and CFN groups. Importantly, small cell clusters or single cells were visualized in the M-CFN treated group, suggesting their considerable inhibition of the self-renewal ability of residual cancer cells. Moreover, to validate the CSC-eradicating effects, we measured the proportion of ALDH-expressing CSCs in tumorspheres from these treatments using flow cytometry analysis (Fig. 3). In 4T1-induced tumorsphere models, the percentage of ALDH^high^ CSCs dramatically decreased from 27.84 ± 3.31% in the control group to 4.61 ± 1.09% in the M-CFN group, resulting in an 83.45% eradication of ALDH^high^ CSCs. Similarly, in MCF-7 tumorsphere models, the proportion of ALDH^high^ CSCs was obviously reduced from 27.0 ± 5.37% in the control group to 7.94 ± 0.40% in the M-CFN group, resulting in a 70.58% eradication of ALDH^high^ CSCs. These data confirmed the more effective CSC-eradicating effects of M-CFN than free CTX and CFN (**p < 0.01), which could be primarily attributed to the potent cytotoxicity of CTX and its effective incorporation into the M-CFN system.

### In vivo tumor accumulation, permeation and CSCs access

**Fig. 4:**
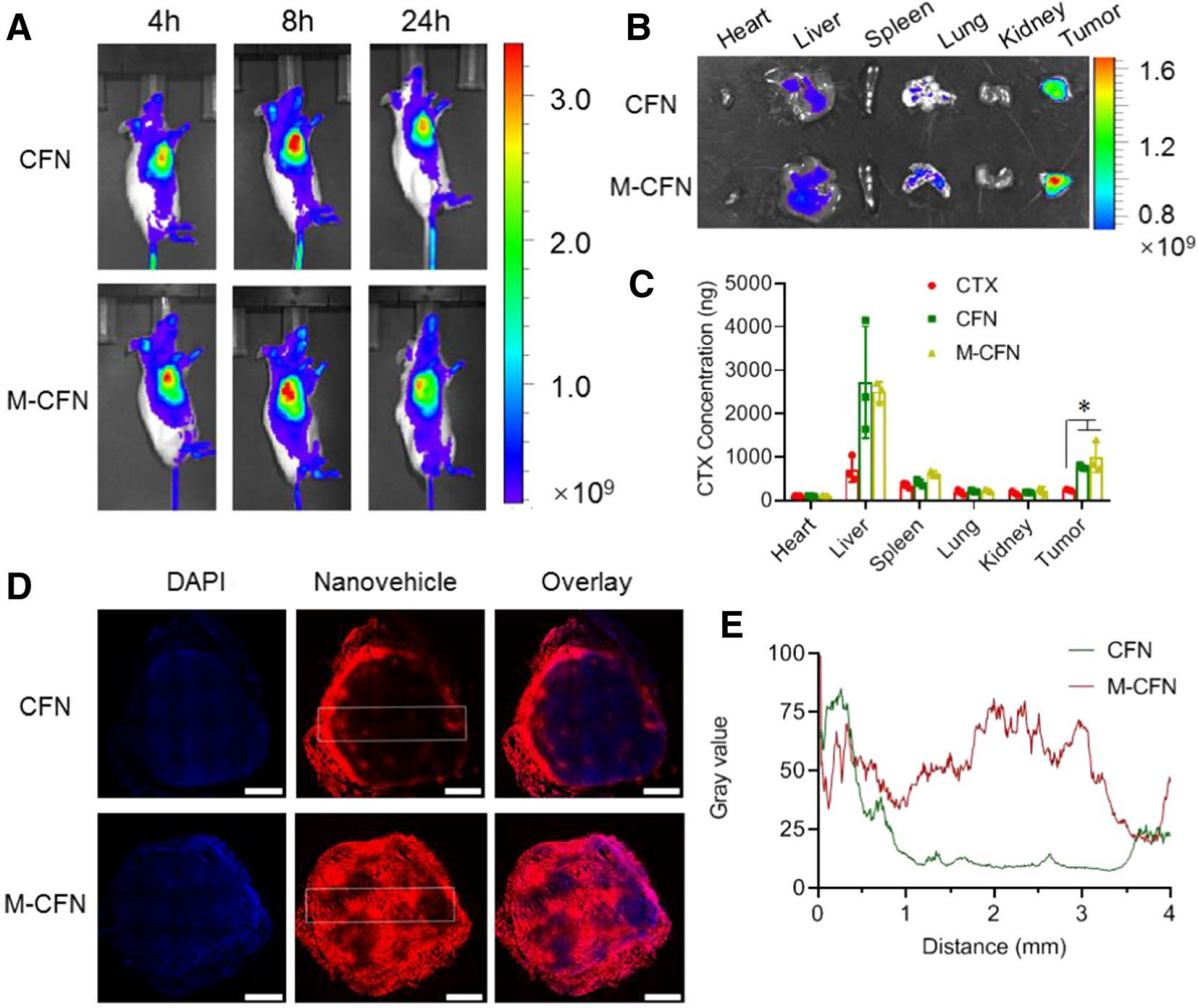
In vivo tumor accumulation and permeation of M-CFN in 4T1 tumor models. **A** In vivo imaging of DiR-labeled CFN and M-CFN at different time points. **B** Ex vivo imaging of DiR-labeled CFN and M-CFN in major organs. **C** Quantified distribution of CTX in major organs from the free CTX-, CFN- and M-CFN-treated groups, *p < 0.05. **D** Intratumor permeation of DiI-labeled nanovehicles, scale bar, 1 mm. **E** Imaging analysis of the intratumor permeation by ImageJ software

The efficient accumulation and deep penetration of nanoparticles in tumor tissues represent a prerequisite for accessing cancer cell and CSC fractions that are frequently located in the deep interior regions of the tumor mass [21, 22, 24, 53]. The in vivo imaging results indicated that the red fluorescence signals of CFN and M-CFN were clearly detected at the tumor sites with high fluorescence intensity (Fig. 4A). The preferential tumor accumulation of CFN and M-CFN was also validated by ex vivo imaging of the major organs (Fig. 4B). At 8.0 h postinjection, CTX levels in tumors from the M-CFN group increased 4.17-fold compared with free CTX but was comparable to that of the CFN group (Fig. 4C). Then, we measured the intratumor permeation of CFN and M-CFN in 4T1 tumors. The red fluorescence signals of M-CFN were extensively visualized in the entire profile of tumor tissue, but those of CFN were only detected in the exterior regions (Fig. 4D). The flexible intratumor permeation of M-CFN was also confirmed by image analysis (Fig. 4E). These data confirmed the notable efficacy of M-CFN in promoting tumor accumulation and intratumor permeation, which was conducive to facilitating their access to the cancer cell and CSC fractions in tumors.

**Fig. 5:**
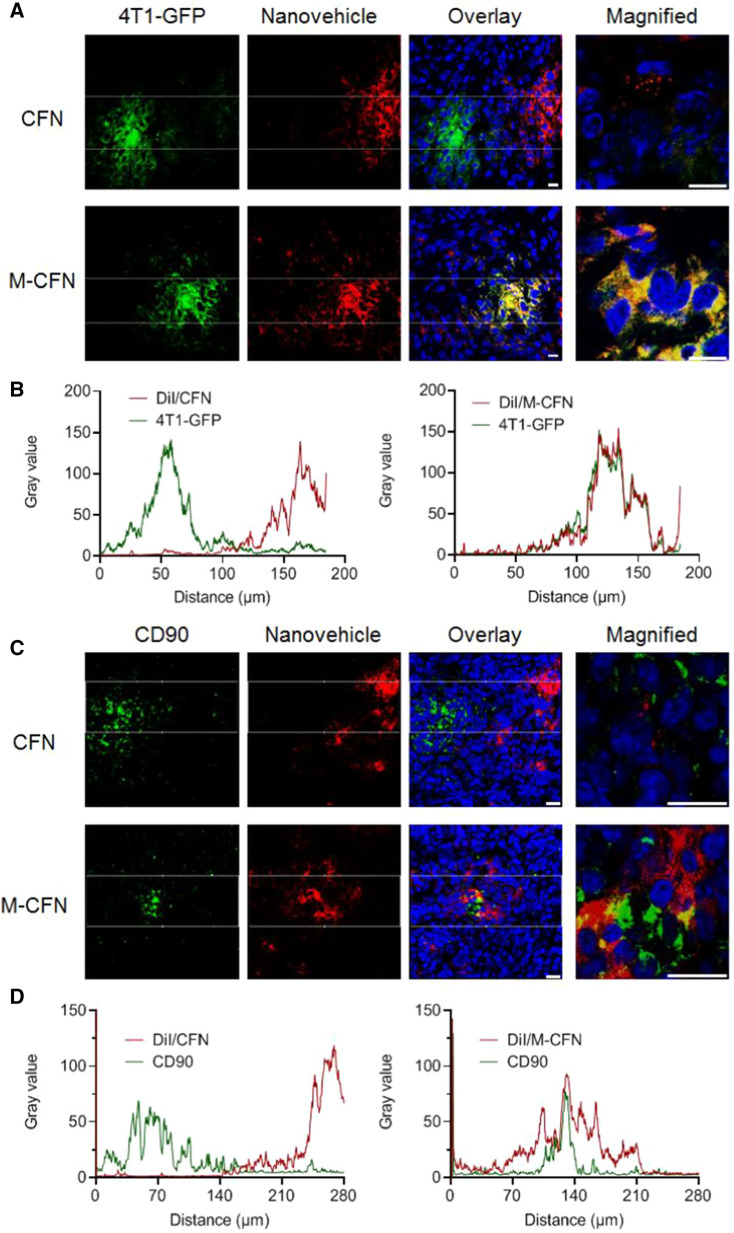
Access of M-CFN to 4T1-GFP cancer cells and CD90-expressing cells in tumors. **A** Access of DiI-labeled nanovehicles to 4T1-GFP cancer cells in tumors and enlarged images, scale bar, 10 μm. **B** Imaging analysis of the cancer cell accessibility of DiI-labeled nanovehicles in tumors. **C** Access of DiI-labeled nanovehicles to CD90-positive cells in tumors and the enlarged images, scale bar, 25 μm. **D** Imaging analysis of the accessibility of DiI-labeled nanovehicles to CD90-positive cells in tumors

Subsequently, we measured the accessibility of these two nanovehicles to the cancer cell fractions in tumors developed from 4T1 cancer cells with stable expression of green fluorescence proteins (4T1-GFP) (Fig. 5). The red fluorescence signals of M-CFN largely overlapped with the green signal-clustered 4T1-GFP regions with high intensity. In contrast, the red fluorescence signals of CFN were primarily localized in regions near the green fluorescence-labeled cancer cell clusters (Fig. 5A). Meanwhile, imaging analysis indicated that the signal profiles of M-CFN and 4T1-GFP cancer cells largely coincided, while those of CFN and 4T1-GFP were two separate peaks with negligible overlaps (Fig. 5B). Meanwhile, the enhanced effects of M-CFN over CFN in accessing the CD90-positive cell fractions in 4T1 tumors were also validated by CLSM imaging and image analysis (Fig. 5C, D). Flow cytometry examinations indicated that uptake of M-CFN in the CD90-expressing cells of 4T1 tumors was considerably enhanced by 2.94-fold in comparison to that in CFN-treated tumors (Additional file 1: Fig. S7). These data effectively confirmed the superiority of M-CFN over CFN in accessing cancer cells and CD90-expressing cells in tumors. By analyzing the formulation of M-CFN and CFN, the advantage of M-CFN in accessing cancer cells and CD90-positive cells in tumors was primarily due to macrophage camouflaging in M-CFN. In addition, the data from the in vitro cellular uptake and in vivo access to CD90-expressing cells effectively confirmed the significance of the CD11b-CD90 interactions in the preferential targeting abilities of the M-CFN particles.

**Fig. 6:**
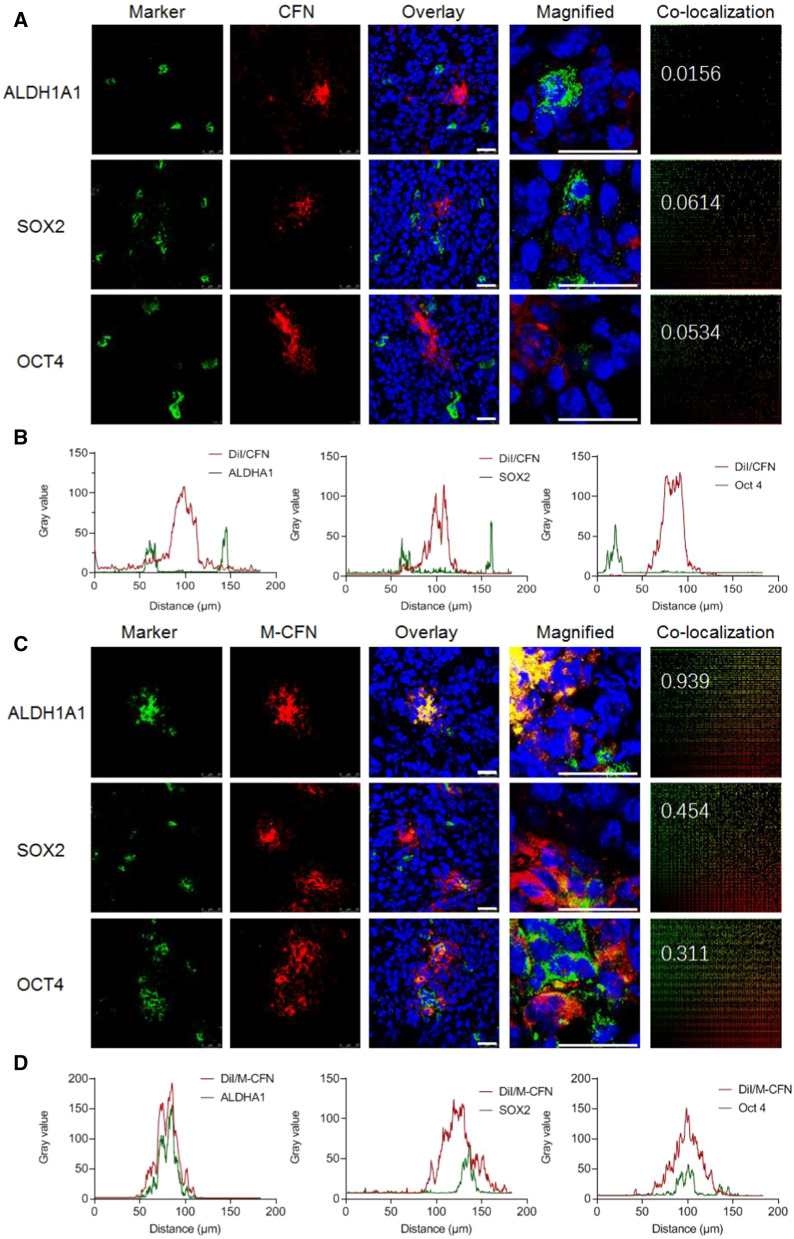
Preferential access of M-CFN to the CSC fractions in 4T1 tumors. **A** CLSM imaging of the access of CFN to the ALDH1A1-, SOX2-, or Oct4-expressing CSC fractions in tumors, the enlarged images, and their colocalization using Image-Pro Analyzer, scale bar, 25 μm. **B** Imaging analysis of the CSCs-accessibility of CFN. **C** CLSM imaging of the accessing of M-CFN to the ALDH1A1-, SOX2-, or Oct4-expressing CSC fractions, the enlarged images, and their colocalization using Image-Pro Analyzer, scale bar, 25 μm. **D** Imaging analysis of the CSCs-accessibility of M-CFN

Importantly, CSCs are a rare subpopulation of cancer cells in heterogeneous tumors that account for the tumor progression and metastasis [3, 7]. The accessibility of M-CFN to the CSC fractions in 4T1 tumors was evaluated using CLSM imaging. ALDH1A1, SOX2 and Oct4 are typical characteristic markers of breast cancer CSCs [7, 53]. To evaluate their CSC-accessing ability, the CSCs in 4T1-induced tumors were labeled with these specific markers by immunofluorescence staining for CLSM detection. As shown in Fig. 6A and C, the red fluorescence signals of M-CFN were considerably colocalized with cells labelled using the specific CSC markers ALDH1A1, SOX2 and Oct4, but the signals of CFN barely merged with these CSC markers. Furthermore, these images were analyzed to confirm the CSC-accessing capacity of M-CFN. The colocalization of M-CFN with ALDHA1-, SOX2- or Oct4-expressing CSC fractions in tumors was significantly higher than that of CFN (Fig. 6A and C). Compared with CFN, the colocalization index of M-CFN with CSCs was significantly increased by 60.19-, 7.39- and 5.82-fold, respectively. Meanwhile, image analysis indicated that the signals of M-CFN largely overlapped with those of the CSCs with specific markers, but those of CFN and CSCs barely colocalized, indicating the preferential access of M-CFN to the CSC fractions in tumors (Fig. 6B and D). Therefore, M-CFN exhibited profound efficiency in promoting intratumor transport and improving accessibility to the cancer cell and CSC fractions in tumors, which was much more efficient than unmodified CFN. Due to the robust physical barriers in tumor tissues, commonly used nanomedicines are usually trapped around the tumor vessels and ineffective penetrating the tumor tissues. The bioinspired design of the M-CFN system could circumvent these obstacles and promote their intratumor delivery. Considering the flexible intratumor permeating ability of macrophage microvesicles and the CD11b-CD90 interactions between TAM and CSC fractions, the macrophage microvesicle-inspired features of the M-CFN system could be the major contributor to the profound CSC-accessing capacity.

### In vivo therapeutic efficacy on tumor growth and metastasis

**Fig. 7:**
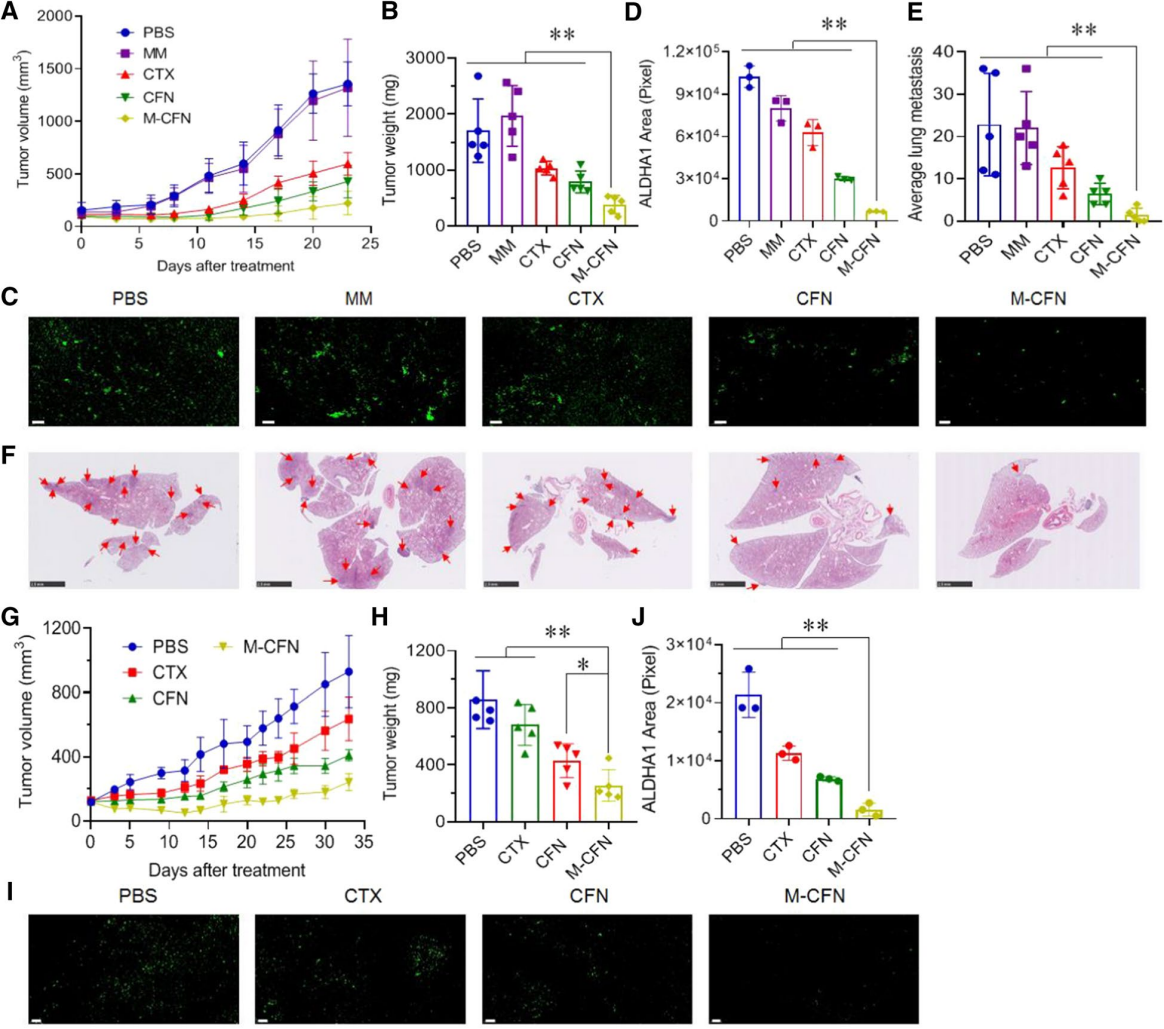
In vivo therapeutic efficacy and eradication of ALDH^high^ CSC fractions in tumors. **A** Tumor growth profiles from each group in 4T1 tumor models (n = 5). **B** Tumor weights from each group in 4T1 tumor models. **C** Effects on eradicating the ALDH^high^ CSCs in 4T1 tumors, wherein the ALDH^high^ CSCs were denoted as green fluorescence signals, scale bar, 50 μm. **D** Quantified effects on eradiating ALDH^high^ CSCs in 4T1 tumors, **p < 0.01. **E** Average number of lung metastatic nodules from each group in 4T1 tumor models, **p < 0.01. **F** Histological examinations of lungs from each treatment in 4T1 tumor models, scale bar, 200 μm. The metastatic foci were denoted as cell clusters with darkly stained nuclei (red arrows). **G** Tumor growth profiles from each group in MCF-7 tumor models (n = 5). **H** Tumor weights from each group in MCF-7 tumor models, *p < 0.05, **p < 0.01. **I** Effects on eradicating the ALDH^high^ CSCs in MCF-7 tumors, wherein the ALDH^high^ CSCs are denoted as green fluorescence signals, scale bar, 50 μm. **J** Quantified effects on eradiating ALDH^high^ CSCs in MCF-7 tumors, **p < 0.01

Encouraged by the prevailing CSC-accessing ability of M-CFN, we finally measured their antitumor efficacy in 4T1-induced and MCF-7 induced breast cancer models (Fig. 7). In the tumor growth profiles of 4T1 metastatic tumor models, tumor progression was inhibited by free CTX, CFN and M-CFN but was mostly unaffected by MM treatment (Fig. 7A). At the final time point, the tumor size in the M-CFN-treated group was only 16.43% of that in the PBS control, producing an 83.57% inhibition of tumor growth. Moreover, the tumor size in the M-CFN group was only 37.38% of free CTX and 52.09% of CFN, indicating the prominent efficacy of M-CFN in inhibiting tumor progression. The notable efficacy of M-CFN on tumor growth was also validated by measuring the weight of primary tumors from each treatment group (Fig. 7B, Additional file 1: Fig. S8). Terminal deoxynucleotidyl transferase-mediated dUTP nick-end labeling (TUNEL) assays revealed the extensive incidence of apoptosis in tumors from the M-CFN-treated group (Additional file 1: Fig. S9). In addition, the body weight was mostly unchanged in response to these treatments (Additional file 1: Fig. S10).

More importantly, we evaluated the effect of M-CFN on eradicating CSC fractions in tumors by immunofluorescence assays, which were denoted as green fluorescence signals. As shown in Fig. 7C, the green fluorescence signals in the M-CFN-treated group were barely detected, and the fluorescence intensity was much weaker than that of the other treatments. The quantified results revealed that the fractions of ALDH^high^ CSCs in tumors in the M-CFN-treated group were significantly reduced by 93.24% compared to the PBS control (Fig. 7D). Moreover, in the M-CFN-treated group, the proportion of ALDH^high^ CSCs in tumors was only 2.91% of that in the free CTX group and 6.09% of that in the CFN group, confirming the superior efficacy of M-CFN in eradicating ALDH^high^ CSCs in tumors. The better CSC-eradicating efficacy of M-CFN over CFN was primarily attributed to its prominent CSC-accessing capacity owing to its macrophage microvesicle-mimicking properties.

Concurrently, we measured the efficacy of M-CFN in suppressing the incidence of lung metastasis. The lungs are one of the most frequent organs of breast cancer metastasis in both preclinical models and clinical patients [54, 55]. Visualized metastatic nodules in the lungs from each group were recorded to evaluate treatment efficacy in inhibiting lung metastasis (Fig. 7E, Additional file 1: Table S1). M-CFN treatment caused 93.86% inhibition of lung metastasis compared to the PBS control. Moreover, the average number of metastatic nodules in the lung from the M-CFN-treated group was only 11.11% that of the free CTX group and 21.88% that of the CFN group. The antimetastatic effects of M-CFN were also confirmed by histological examinations of the metastatic foci in lungs from each treatment (Fig. 7F). These results suggested the prevailing efficacy of M-CFN in suppressing the incidence of lung metastasis, which could be related to the profound eradication of CSC fractions in the primary tumor sites.

To further confirm the efficacy of M-CFN, we measured the therapeutic effects on inhibiting tumor growth and eliminating CSCs in human MCF-7 induced tumor models (Fig. 7G–J). In the tumor growth profiles, tumor size was moderately inhibited by free CTX and CFN but significantly retarded by M-CFN treatment (Fig. 7G). The body weight of each group rarely changed during these treatments (Additional file 1: Fig. S11). At the final time point, tumor size in the M-CFN-treated group decreased by 73.91% compared to the PBS control and was much lower than that in the free CTX and CFN groups. The tumor inhibitory effects of M-CFN were also validated by measuring the tumor weights from each treatment (Fig. 7H, Additional file 1: Fig. S12). Meanwhile, M-CFN treatment produced the best efficacy in reducing the ALDH^high^ CSC fractions in tumors (Fig. 7I). Compared with the PBS control, M-CFN treatment caused a 92.52% reduction in ALDH^high^ CSC fractions in MCF-7 tumors (Fig. 7J). Moreover, the proportion of ALDH^high^ CSCs in tumors from the M-CFN group was only 14.11% of free CTX and 23.20% of CFN. As a result, M-CFN treatment produced notable efficacy in suppressing tumor growth and eliminating the ALDH^high^ CSC fractions in MCF-7 tumors. From these data, it can be deduced that the bioinspired design of the M-CFN system could effectively combine the advantages of the nanotool delivery system and the tumor-tropic features of M2 macrophages and demonstrated encouraging potential for targeting the cancer cell and CSC fractions in tumors for effective cancer treatments. However, due to the possible immunogenicity of macrophages from a different donor or species, this design of macrophage microvesicle-inspired nanovehicles may be limited to autologous macrophages. In addition, in view of the high expression of some immune checkpoints on the M2 macrophage surface [44], their impacts on cancer immunotherapy will be considered in our future studies.

## Conclusions

In summary, we successfully designed a M2 macrophage microvesicle-inspired nanovehicle of cabazitaxel (M-CFN) by camouflaging macrophage membranes onto CFN to preferentially access cancer cells and CSCs in tumors for cancer therapy. In the 4T1 tumor model, M-CFN effectively permeated across the tumor mass and accessed both the cancer cell and CSC fractions. Moreover, M-CFN effectively promoted accessibility to the ALDHA1-, SOX2- or Oct4-expressing CSC fractions in tumor. Importantly, M-CFN treatment profoundly eliminated the ALDH^high^ CSC fractions in 4T1- and MCF-7-induced tumors and produced notable therapeutic benefits in these two breast cancer models. Therefore, M2 macrophage microvesicle-inspired nanovehicles represent an encouraging bioinspired drug delivery nanoplatform for permeating tumor tissues and accessing cancer cells and CSCs in tumors for effective cancer therapy.

## Supplementary Information

**Additional file 1. **Additional data, including materials and methods, materials synthesis and characterizations, in vivo therapeutic efficacy on tumor growth and lung metastasis.

## Data Availability

The data supporting the findings of this study are available within this paper and Additional files. Additional data can also be available from the corresponding author on reasonable request.

## Abbreviations

CSCs: Cancer stem cells
M-CFN: M2 macrophage microvesicle-inspired nanovehicle of cabazitaxel
ALDH: Aldehyde dehydrogenase
CAFs: Cancer associated fibroblasts
TAMs: Tumor-associated macrophages
ECM: Extracellular matrix
CTX: Cabazitaxel
CFN: Cabazitaxel-loaded polyfluorocarbon nanoparticles
PFP: Polyfluorocarbon polymeric
PCL-PEG: Poly(ε-caprolactone)-block-poly(ethylene glycol)
MM: Macrophage membrane
CSF1R: Colony stimulating factor 1 receptor
CCR2: C–C motif chemokine receptor
EE: Encapsulation efficiency
HPLC: High performance liquid chromatography
PBS: Phosphate buffered saline
CLSM: Confocal laser scanning microscopy
4T1-GFP: 4T1 cancer cells with stable expression of green fluorescence proteins
TUNEL: Terminal deoxynucleotidyl transferase-mediated dUTP nick-end labeling
