# Supplementary material for: M2 macrophage microvesicle-inspired nanovehicles improve accessibility to cancer cells and cancer stem cells in tumors
Source: J Nanobiotechnology. 2021 Nov 27;19:397. doi: 10.1186/s12951-021-01143-5 (PMC8627085; doi:10.1186/s12951-021-01143-5)
Supplement: Supplementary file 1 — Additional file 1. Additional data, including materials and methods, materials synthesis and characterizations, in vivo therapeutic efficacy on tumor growth and lung metastasis. [file 12951_2021_1143_MOESM1_ESM.doc]

**Additional file 1**

**M2 macrophage microvesicle-inspired nanovehicles improve accessibility to cancer cells and cancer stem cells in tumors**

Yuqi Wang^1, 2^, Xiang Gong ^2^, Jie Li ^2^, Hong Wang ^2^, Xiaoxuan Xu ^2^, Yao Wu ^2^, Jiaoying Wang ^2^, Siling Wang ^1, *^, Yaping Li ^2, *^, Zhiwen Zhang ^2, 3, *^

^1^ School of Pharmacy, Shenyang Pharmaceutical University, Shenyang 110016, Liaoning, China.

^2^ State Key Laboratory of Drug Research & Center of Pharmaceutics, Shanghai Institute of Materia Medica, Chinese Academy of Sciences, Shanghai 201203, China

^3^ Yantai Key Laboratory of Nanomedicine & Advanced Preparations, Yantai Institute of Materia Medica, Shandong, 264000, China.

*****Correspondence author: Prof. Siling Wang (silingwang@syphu.edu.cn), Prof. Yaping Li ([ypli@simm.ac.cn](mailto:ypli@simm.ac.cn)) or Prof. Zhiwen Zhang (zwzhang0125@simm.ac.cn)

Additional Results


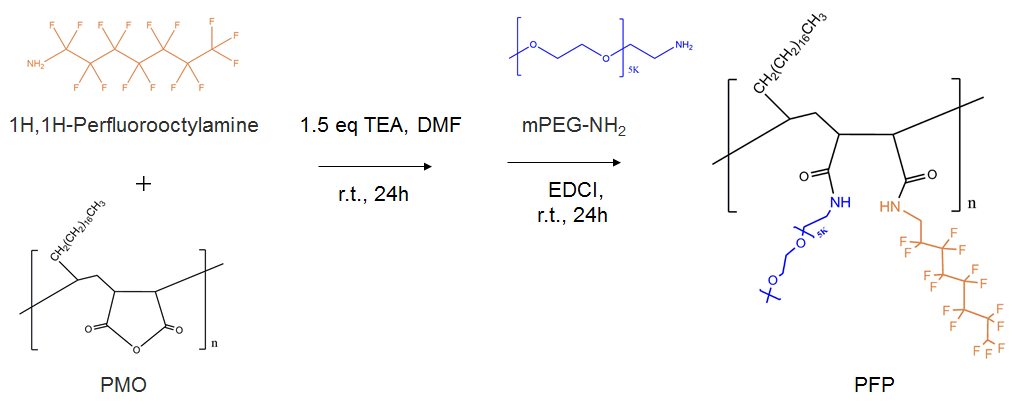


**Fig. S1** Synthesis of PFP. PFP was synthesize by grafting 1H,1H-perfluorooctylamine (57 mg) and mPEG-NH_2_ (714 mg) to PMO (50 mg). In brief, the 1H,1H-perfluorooctylamine and PMO were dissolved in dimethyformamide (DMF) with 1.5 eq of triethylamine (TEA) and kept at room temperature (r.t.) for 24 h. Then, the mPEG-NH2 with 1.0 eq of 1-ethyl-3-(3-dimethylaminopropyl) carbodiimide hydrochloride (EDCI) were added to the mixed solution and maintained at r.t. for further 24 h. The reaction solution was purified by a dialysis method (molecular weight cut-off, 12 kDa) and freeze-dried to obtain PFP for further characterization.


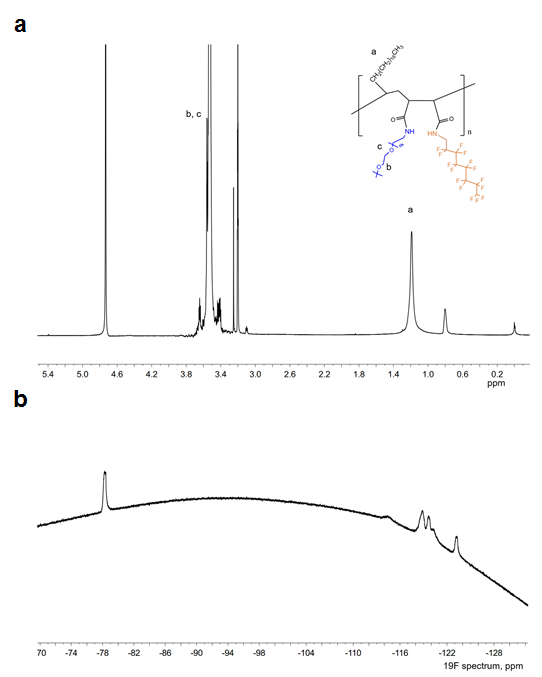


**Fig. S2** Characterization of PFP by ^1^H-NMR (a) and Fluorine spectra (b).


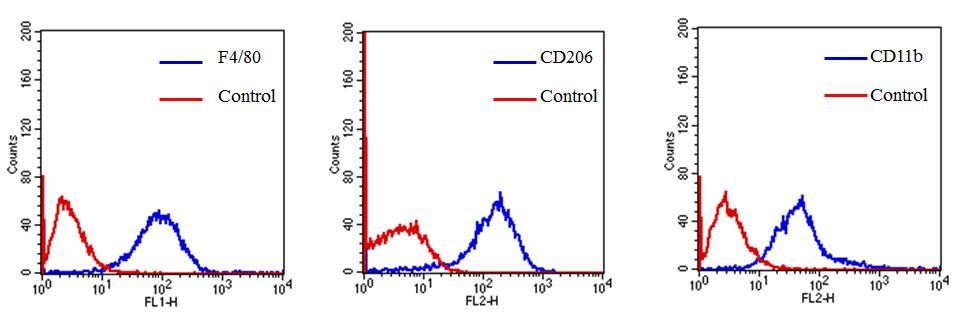


**Fig. S3** Flow cytometer analysis of M2 macrophage with high expression of F4/80, CD206 and CD11b.

**Fig. S4** The particle size changes of CFN and M-CFN upon their incubation in PBS (pH 7.4) at room temperature


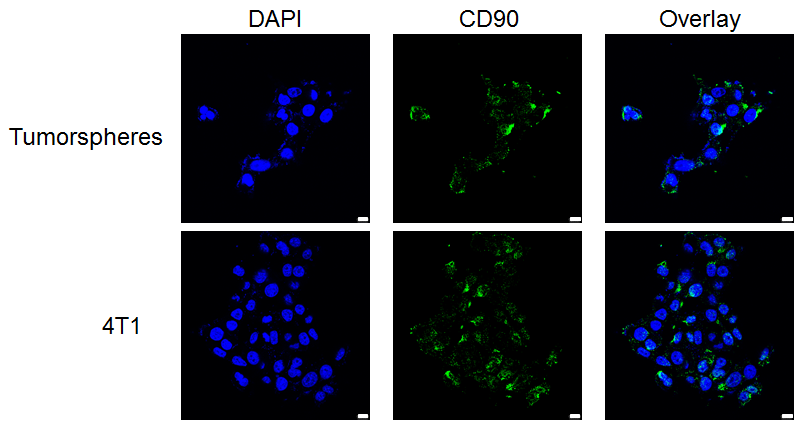


**Fig. S5** Expression of CD90 in CSCs-enriched 4T1 tumorspheres and parent 4T1 cancer cells by immunofluorescence assays, scale bar, 10 µm.

**Fig. S6** Cytotoxicity of free CTX, CFN and M-CFN in 4T1 cancer cells.


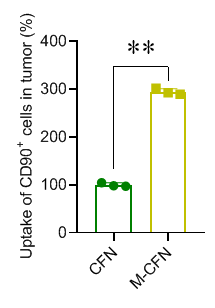


**Fig.** **S7** Access of CFN and M-CFN to CD90-positive cells in 4T1 tumors

tumors.
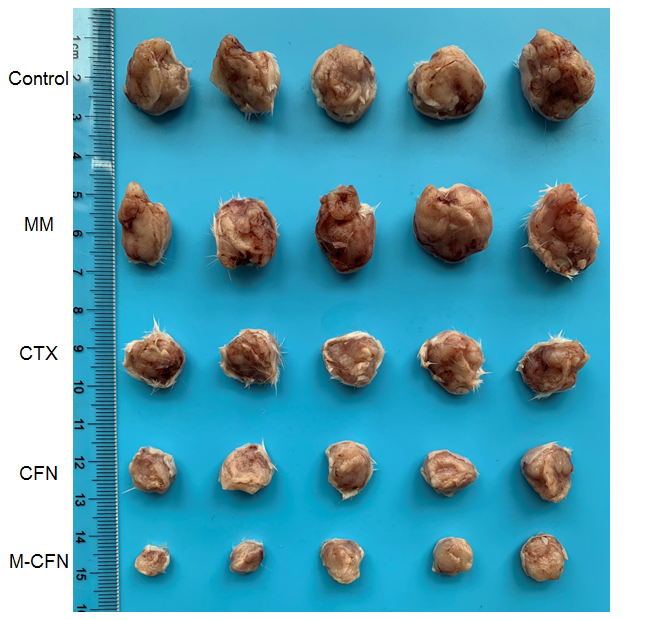


**Fig. S8** Typical photographs of tumors from each treatment in 4T1 tumor models.


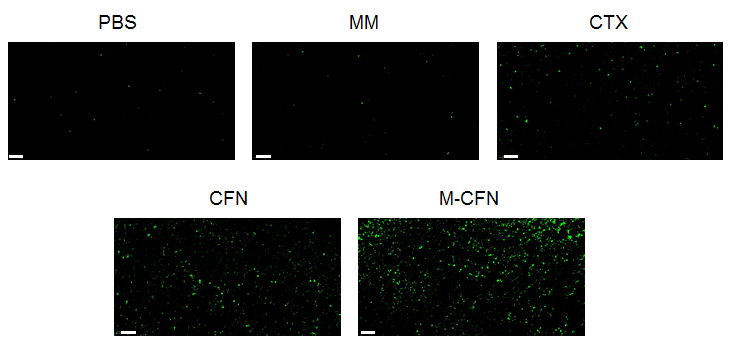


**Fig. S9** TUNEL staining of the tumors from each treatment, wherein the damaged cells were denoted as green fluorescence signals, scale bar, 50 μm.

**Fig. S10** Body weight changes of 4T1 tumor-bearing mice from each treatment.

**Fig. S11** Body weight changes of MCF-7 tumor-bearing mice from each treatment.


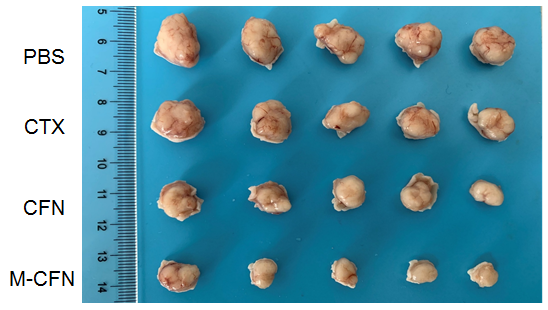


**Fig. S12** Typical photographs of MCF-7 tumors from each treatment.

**Table S1** The number of metastatic nodules per lung in 4T1-indcued metastatic breast cancer models from each treatment.

| Group | Number of metastatic nodules per lung | | | | | Mean | SD |
| --- | --- | --- | --- | --- | --- | --- | --- |
| PBS | 36 | 12 | 20 | 35 | 11 | 22.80 | 12.11 |
| MM | 36 | 18 | 20 | 23 | 13 | 22.00 | 8.63 |
| CTX | 9 | 18 | 14 | 16 | 6 | 12.60 | 4.98 |
| CFN | 7 | 4 | 7 | 10 | 4 | 6.40 | 2.51 |
| M-CFN | 4 | 1 | 0 | 2 | 0 | 1.40 | 1.67 |
